# Case of Vesico-Vaginal Fistula and Laceration of Anterior Perineum

**Published:** 1876-06

**Authors:** A. B. Newkirk

**Affiliations:** Hyde Park, Ill.


### A CASE OF VESICO-VAGINAL FISTULA AND LACERATION OF ANTERIOR PERINEUM,

### THE DIRECT RESULT OF THE MALADROIT USE OF FORCEPS.

By A. B. NEWKIRK, M.D., Hyde Park, Ill.

Mrs. Maria H. was a native of England, 35 years of age; five feet eight inches in height; weight, 180 lbs.; mother of four children, and of robust health up to the time of her last accouchement, July 27th, 1875.

Mrs. H.'s first three children were born with easy natural labors, the first child weighing 12 pounds, and the second and third 10 pounds each at birth, and the last child, with an unusually small round head, though not weighed, was supposed to weigh betwen 6 and 7 pounds.

Mrs. H.'s last labor commenced about four o'clock in the morning and continued with slight increase in the force and frequency of the pains until ten o'clock, when Dr. J. (homœopathist), who having been called was in attendance, expressed his unwillingness to wait longer for more forcible pains, proceeded to deliver with forceps, and had great difficulty in applying them, "owing to a very rigid condition of the uterus," and in their application and use caused great pain and suffering to the patient, the forceps slipping seven or eight times. The Dr. continued the use of the forceps for about four hours, excepting that when he would become fatigued from protracted effort, he would rest for a few minutes at a time.

Delivery was finally effected about two o'clock P. M., of a male child with scalp lacerated, much bruised and tumefied on the antero-superior temporal region, where there is now to be seen an indelible cicatrix, and the left eye and cheek bruised and swollen and the latter lacerated. The delivery was followed by alarming hæmorrhage.

On the day following delivery there was a great deal of soreness, pain and tumefaction of the genitals, and *the urine no longer flowed through the urethra, but*

*passed through the vagina*, and continued to do so up to the time of the death of the patient. The inflammatory action set up in the vagina and pelvic viscera (evidently by the maladroit use of the forceps) continued for about four months, with resultant constitutional disturbance of varying intensity, until December 3rd, when there was a discharge at one time of over half a gallon, by measure, of very offensive muco-purulent matter, after which the sufferings of the patient were much less. Mrs. H. continued during these four months under the care and treatment of Dr. J., who, during this time contributed greatly to the discomfort and suffering of the patient by the caustic and other local applications made by him to relieve the immediate effects of the first injury, until she became greatly anæmic after her long suffering. Then Dr. J., discouraged, perhaps, by the results of his blunders, informed the husband of the suffering woman, Mr. John H., an intelligent and accomplished florist and gardener, that his wife was laboring under cancer of the womb; that he could do nothing more for her, except with a view to mitigate her suffering, and that she would linger for three years with the cancer.

Mr. H. was much grieved when informed that his wife had cancer of the womb, as he had lost a former wife in consequence of this affection. Although a poor man, he was anxious to have the sufferings of his wife, as far as possible, mitigated if not relieved, and hence sought and obtained advice and treatment by several physicians without benefit.

On Jan. 21st, last, I was called to see the patient, and obtained from her, her husband, and the nurse who was present at the time of her accouchement and for some time thereafter, the above narrated history of this case, substantially. I found Mrs. H. an object of pity and compassion; her cadaverous face and anæmic condition impressed me with the belief that she might not survive the night without the liberal use of stimulants and cordials. She was certainly too weak to be removed from where she lay upon a lounge remote from a suitable light in order to undergo an examination. I therefore directed that she be given freely of stimulants and beef tea, and took my leave, promising to return the following morning, and, if the condition of the patient then would justify it, I would complete the examination of the case.

The following morning I found her more comfortable and somewhat refreshed by the stimulants and beef tea ordered the previous day, and proceeded to carefully examine the parts, after placing her upon a table in front of a good light, and rinsing the vagina well with a solution of carbolic acid.

I found as lesions, vesico-vaginal fistula two inches in length antero-posteriorly and one inch in its greatest transverse diameter in the bas-fond of the bladder ; its edges were smooth and somewhat thickened. The internal surface of the bladder was quite corrugated in appearance, and its cavity of not more than one-half the normal size.

I could see, while looking through the speculum and fistulous opening, the urine dropping at regular intervals from the ureters into the bladder.

The canal of the urethra was closed by viscid mucus (or adhesions), so as to require considerable force to introduce a catheter.

I could not see the os uteri, but it, with that portion of the uterus presented to view, felt firm and immovable, apparently in consequence of adhesions of its posterior face to contiguous tissues.

There were several ulcerated surfaces to be seen upon the mucous membrane lining the vagina, one on its posterior face fully an inch in its greatest diameter, and extending in depth nearly or quite through it.

The anterior perineum and about two inches in length of the inferior recto-vaginal septum, with its mucous covering, were lacerated, leaving the rectum for that distance exposed anteriorly.

The lacerated and ulcerated surfaces were discharging a muco-purulent matter, freely.

The extent of the consequent drain upon the system may be approximated when we consider that, during the examination, lasting about thirty minutes, there was collected upon a napkin placed under the hips of the patient, about a fluid ounce, notwithstanding the precaution taken, before commencing the examination, of rinsing well the vagina and bladder with a solution of carbolic acid.

I inquired of the patient particularly, *if she had had any lesions of the bladder or perineum before her last confinement, and, at what time she first noticed any; also, when she first noticed the urine pass through the vagina;* to which she replied, that "she was perfectly healthy and had no trouble of the kind named before her last babe was born; that *the day after that,* she noticed that her water did not pass out the natural way, but dribbled away from her continually through the 'birth place;' that the 'birth place' was so inflamed and painful to her for some time that she could not bear to have it touched; and when the swelling somewhat subsided, she found the lower part of the outlet all torn away; that the doctor nearly killed her with his instruments when she was confined, for he hurt her so badly when he was fixing them and when they slipped, as they did seven or eight times."

After completing the examination of the case and satisfying myself as to the condition of the patient, I explained to Mrs. H. and her husband, the nature of the case, (the first intimation of the kind they had received from any one), probable cause, and results to be reasonably expected from treatment; that vesico-vaginal fistula could be produced in three ways: first, by idiopathic disease of the bladder, or agents acting mechanically upon its internal surface, resulting in lesions, first of its inferior walls, and secondly of the superior walls of the vagina; second, that a child's head, relatively large, impacted in the superior strait of the pelvis, and so pressing upon the neck of the bladder sufficiently long, would result in inflammation and sloughing of its walls and that of the vagina; third, by direct mechanical means operating from within the vagina, first upon its superior walls, and secondly upon the inferior wall of the neck or bas-fond of the bladder.

In carefully examining the history of this case, as presented above, we find no evidence of the first named cause having existed *prior* to the existence of the fistulous opening.

When we consider the fact of the comparatively large and well developed pelvis of the mother, and the fact of the child's head being round, and small for a child weighing less than seven pounds, and the further fact that there is no evidence going to show that there was any retention of the head in contact with the bladder by impaction, and the additional fact, that, if produced by long pressure of the head upon the bladder the resulting fistulous opening would not have been produced so soon as it was in this case by several days, we are justified in the conclusion that in this case the fistulous opening cannot be the result of the second cause named.

We are, therefore, left with the third cause named to account for the lesion of vesico-vaginal fistula, and by the same course of reasoning we are forced to conclude that the lesions of the antero-perineum in this case were produced by the same cause, to wit, the maladroit use of forceps, perhaps when they slipped, as the patient states they did seven or eight times, producing great pain and suffering at the time, their edge coming in contact with the tense perineum with considerable force while traction was being made, or it may have been made in a line with the axis of the superior strait of the pelvis.

I suggested to Mr. H. the advisability of calling other members of the profession to see the case, as it was one of considerable interest, to which he readily assented, and A. H. Mann, M.D., of South Chicago,

J. E. Morrison, M.D., of Hyde Hark, J. Ramsay Flood, M.D., Assistant Physician to the Woman's Hospital of the State of Illinois, and A. Reeves Jackson, M.D., Surgeon-in-Chief of the same institution, were called in consultation and made a careful examination of the case, and fully confirmed the views above expressed by myself as to the nature and probable cause of the existing lesions, and the results of treatment to be reasonably expected in the case.

I continued the treatment of Mrs. H. up to the time of her death, with results somewhat variable during this period.

For the first two months, a generally tonic and hygienic treatment, varied from time to time as circumstances required, was upon the whole found to be beneficial; the patient being able to sit up on an easy chair for a while each day of the last week of the time.

On March 24th, the condition of the patient became less favorable by reason of the supervention of enteritis, which continued, notwithstanding the remedies used to avert it, until April 10th, when she died.

I made a post-mortem examination of Mrs. H. on the following morning, with the assistance of Charles Adams, M.D., and J. E. Morrison, M.D., in the presence of Mr. H. and Dr. J. We found the small intestines extensively inflamed, and the transverse portion of the duodenum prominently so. The mesenteric glands and glands of Peyer, with the right kidney, were also inflamed. We found the uterus by the lower two-thirds of its posterior face firmly attached by adhesions to contiguous tissues, and that portion of its body below its peritoneal covering soft and friable, so that in the effort to separate the above named adhesions with the fingers, the superior third and anterior face of the body of the womb readily separated from the adherent portions.

We found the bladder of about half the normal size, with fistulous opening in its base of one by two inches in diameter.

